# *Anopheles* (*Anopheles*) *petragnani* Del Vecchio 1939—a new mosquito species for Germany

**DOI:** 10.1007/s00436-016-5014-5

**Published:** 2016-03-22

**Authors:** Norbert Becker, Wolf Peter Pfitzner, Christina Czajka, Achim Kaiser, Thomas Weitzel

**Affiliations:** German Mosquito Control Association (KABS), Institute for Dipterology, Georg-Peter-Süß-Str. 3, 67346 Speyer, Germany; Ruprecht-Karls-Universität Heidelberg, Im Neuenheimer Feld 230, 69120 Heidelberg, Germany

**Keywords:** *Anopheles petragnani*, Anopheles Claviger Complex, Rock pools, Chaetotaxy, ITS2

## Abstract

The so far known species of the Anopheles Claviger Complex, *Anopheles claviger* s.s. and *Anopheles petragnani*, can only be distinguished by partial overlapping characteristics of immature stages and by nucleotide sequence variation of the genomic ribosomal DNA (rDNA) internal transcribed spacer 2 (ITS2) region. The known distribution of *An. petragnani* is so far restricted to the western Mediterranean region, whereas *An. claviger* s.s. occurs across most of Europe, up to the Middle East and North Africa. In our study, we investigated the larval mosquito fauna in rock pools of the Murg valley (Black Forest, Germany) once a month from April to December 2015.

Among other species, larvae belonging to the Anopheles Claviger Complex were found. The fourth instar larvae were morphologically identified by chaetotaxy of the head and abdomen. The results were confirmed by a multiplex PCR and additional sequencing of the amplificates.

Of the 1289 collected larvae from the rock pools, seven belonged to the Anopheles Claviger Complex. Five individuals were determined morphologically as *An. petragnani* and two as *An. claviger* s.s. The associated mosquito fauna comprised of *Aedes japonicus japonicus* (548 individuals), *Culex pipiens* s.l. and *Culex torrentium* (493 individuals) and *Culex hortensis* (241 individuals).

This is the first record of *An. petragnani* north of the Alps. Further studies will reveal whether this is an isolated population of *An. petragnani* and if the investigated rock pool breeding sites represent typical habitats of this species in temperate regions in Central Europe.

## Introduction

*Anopheles* (*Anopheles*) *claviger* s.s. (Meigen, 1804) and *Anopheles* (*Anopheles*) *petragnani* Del Vecchio, 1939 are sibling species, forming the Anopheles Claviger Complex, as males and females cannot be distinguished by morphological characteristics. However, both species differ considerably in their egg, larval and pupal morphology, as well as in their adult behaviour. *An. petragnani* was first described by Del Vecchio in 1939 as a variety of *An. claviger*, distinguished by the shape of the eggs (Del Vecchio 1939). Coluzzi highlighted the larval and pupal differences between these forms and proved, using cross-mating studies, that they are two distinct species (Coluzzi 1960; Coluzzi 1962). Since then, *An. petragnani* has only been found in the western Mediterranean area, mainly in coastal regions (Ramsdale and Snow 2000). The larvae usually develop along river beds in fresh water rock pools or in ditches and drainage canals (Marchi and Munstermann 1987).

*An. claviger* s.s. is a Palaearctic species that is distributed all over Europe, the Middle East and North Africa (Ramsdale and Snow 2000). The larvae are found in a wide variety of breeding sites, but in general occur in unpolluted, semipermanent and permanent water bodies (Becker et al. 2010). They are more adapted to colder water temperatures than *An. petragnani*, which results in a wider distribution area towards the North. In warmer, Mediterranean regions, *An. claviger* s.s. exploits colder breeding sites such as wells and underground water bodies (Coluzzi 1962; Ramsdale and Snow 2000). In the geographic range of *An. petragnani*, both species are sympatric and can also be associated in the same breeding sites (Coluzzi 1960). In the Upper Rhine Valley in southwest Germany, *An. claviger* s.s. is a common mosquito species and can be found regularly (Becker and Kaiser 1995).

Little is known about rock pool mosquito species diversity in Germany. Only Vogel (Vogel 1933) has reported on the occurrence of *Culex* (*Maillotia*) *hortensis* Ficalbi, 1889 and *Culiseta* (*Culiseta*) *glaphyroptera* (Schiner, 1864) in rock pools in the Murg valley, Black Forest. Therefore, in 2015, a monitoring programme was initiated to survey the occurrence of rock pool mosquitoes in this region, primarily with a special emphasis on *Aedes* (*Hulecoeteomyia*) *japonicus japonicus* (Theobald, 1901). This survey led to the recording of *An. petragnani* larvae in the investigated rock pools of the river Murg in August 2015.

After this first detection of *An. petragnani*, a study on the larval morphology of both sibling species belonging to the Anopheles Claviger Complex was initiated. The species differentiation in larval seta distribution (Coluzzi 1960; Coluzzi et al. 1965) was analysed and verified using a specific PCR assay (Kampen et al. 2003; Sternberg 2004) in combination with sequencing of the nuclear ribosomal internal transcribed spacer 2 (ITS2) region.

## Method

### Mosquito origin

**Fig. 1:**
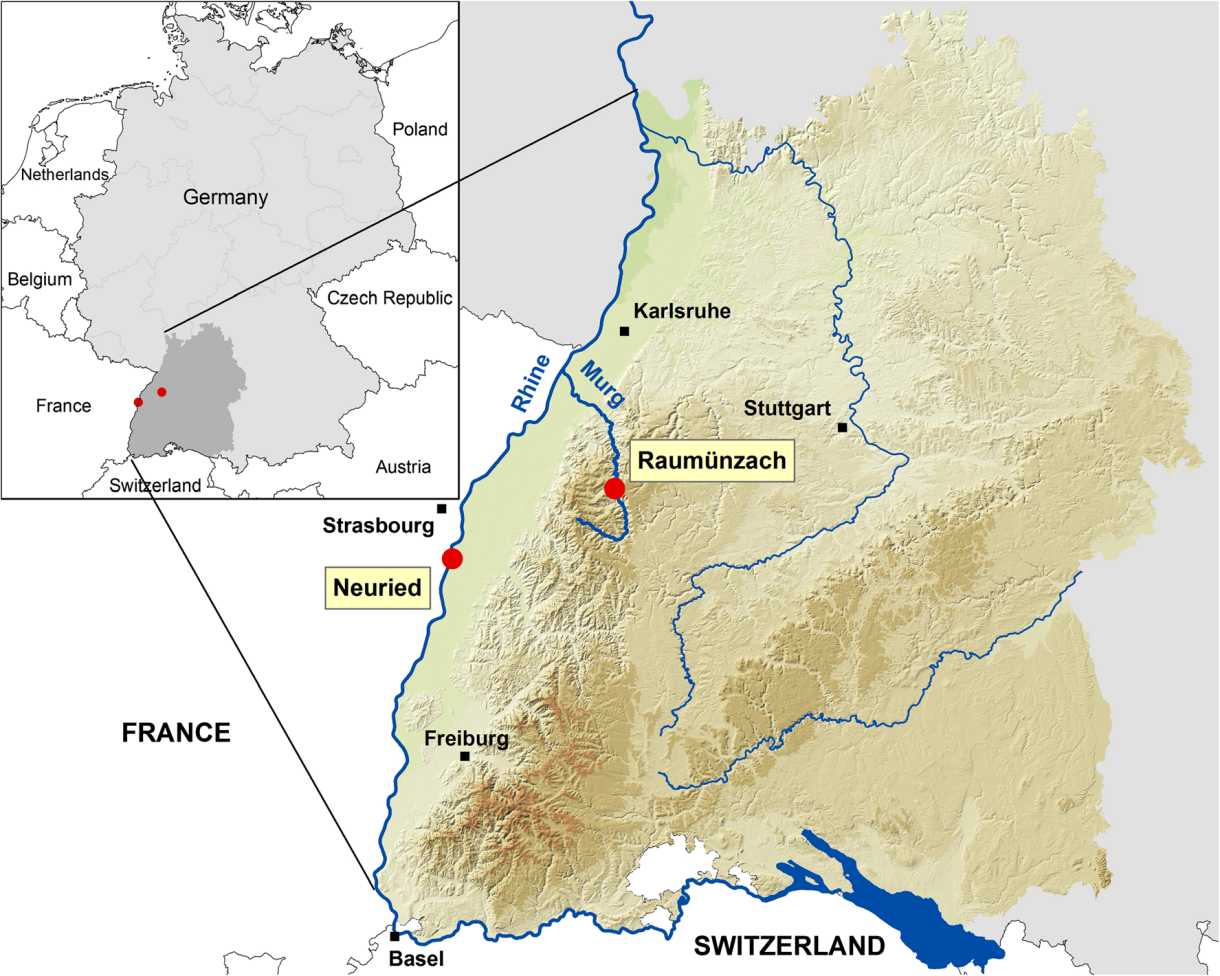
Map of Baden-Wuerttemberg, Germany. The *red dots* indicate the trapping sites at the river Murg in the Black Forest (Raumünzach) and in the flood plains of the river Rhine (Neuried)

Twelve rock pools in the Murg valley at Raumünzach (Black Forest, 48.633 N, 8.356 E, 420 m a.s.l.; Germany, Fig. 1) were monitored once a month from April to December 2015. In each breeding site and at each sampling, 10 dips with a WHO standard dipper (350 ml) were taken and sieved through a net. The larvae were reared to the fourth instar in the lab and transferred into vessels with 70 % ethanol for species determination and assessment of the species composition.

For morphological comparison, additional larvae belonging to the Anopheles Claviger Complex were collected in September 2015 in ditches with little vegetation in the floodplains of the river Rhine near the municipality of Neuried (48.459 N, 7.772 E; 145 m a.s.l.) (Fig. 1).

### Abiotic parameters of the breeding sites

The abiotic parameters, pH value, conductivity, water temperature, carbonate hardness, total hardness, ammonium, nitrite and nitrate, as well as phosphate, were determined at each sampling date by employing the following test sets: pH using a pocket pH metre (Amarell Electronic), conductivity and temperature (Hanna instruments, Australia, HI98129) and all other parameters with the Compact Laboratory for Water Testing (Merck 1.11151.0001).

### Morphological identification

The fourth instar larvae were morphologically identified according to the key of Becker et al. (2010). To improve determination, some larvae were mounted in Euparal. The diagnostic characters used to distinguish *An. claviger* s.s. from *An. petragnani* were the number of branches of the postclypeal setae (4-C), as well as the number of branches of the antepalmate setae of the abdominal segments IV and V (2-IV and 2-V). According to Becker et al. (2010), the postclypeal setae of the fourth instar larvae of *An. claviger* s.s. possess two to five branches and the antepalmate setae three to five branches. In *An. petragnani*, the postclypeal setae are usually single, but sometimes with two branches, and the antepalmate setae have one to three branches.

### Molecular identification

After morphological classification, the anopheline larvae were determined using a PCR of the second internal transcribed spacer (ITS2) region of mosquito ribosomal DNA (rDNA). Therefore, DNA from all the collected specimens was extracted with QuickExtract™ DNA Extraction Solution 1.0 (epicentre, Madison, WI, USA), following the manufacturer’s manual, and kept frozen at −20 °C until further use.

PCR analysis was performed with the universal f-primer 5.8S (5′-TGTGAACTGCAGGACACATG-3′) and two species-specific r-primers: 5′-GCAACACTTTGGTGGCCAC-3′ (*An. petragnani*) and 5′-CTAGCAAGTGCACTGTGTCC-3′ (*An. claviger* s.s.) (Kampen et al. 2003; Sternberg 2004). The amplificates of 367 bp (*An. petragnani*) and 269 bp (*An. claviger* s.s.), respectively, within the ITS2 sequence reliably distinguish both species.

The PCR mixture was made up of 10 μl 5× Phire Green Reaction Buffer (containing 1.5 mM MgCl_2_ at the final 1× reaction concentration), 1 μl Phire Hot Start II DNA-Polymerase (20 mM Tris-HCl (pH 7.4 at 25 °C), 0.1 mM EDTA, 1 mM DTT, 100 mM KCl, stabilisers, 200 μg/ml BSA, 50 % glycerol) (both Thermo Scientific, Waltham, MA, USA), 1 μl dNTPs (200 μM each), 1 μl of each primer (200 nM), and 2 μl DNA solution and water, adding up to a total reaction volume of 50 μl. For the negative control, 2 μl of water was used instead of DNA.

The cycling protocol began with an initial denaturation step of 30 s at 98 °C, followed by 30 cycles, consisting of denaturation at 98 °C for 5 s, annealing at 52 °C for 45 s, as well as elongation for 15 s at 72 °C, and ended with a final elongation step at 72 °C for 60 s.

From each PCR-product, 5 μl was loaded on a 1.2 % agarose gel in a FlashGel™ System (Rockland, ME, USA). Electrophoresis was run for 10 min at 200 V to verify the species-specific size of the amplicons.

All amplificates of *An. petragnani* and a representative number of amplificates of *An. claviger* s.s. were further prepared for sequencing, using the GF-1 PCR Clean-up Kit, according to the manufacturer’s instructions (Vivantis, Oceanside, CA, USA). The sequencing reactions were carried out by Eurofins Genomics (Ebersberg, Germany).

## Results

### Species composition

In two of the 12 analysed Murg rock pools, seven anopheline larvae were sampled in August and September 2015. Four of them were identified by morphological and molecular methods as *An. petragnani* and two of them as *An. claviger* s.s. A single male, hatched from the pupal stage, was also determined as *An. petragnani*, however only by PCR.

In the floodplains of Neuried, only *An. claviger* s.s. was found. All 20 larvae were determined morphologically, whereas 15 were additionally confirmed by PCR.

The total mosquito fauna in the rock pools was assayed by 1050 dips and included 1289 mosquito larvae. The exotic species *Ae. j. japonicus* was represented by 548 larvae (42.5 %). Four hundred ninety three larvae (38.2 %) were determined as *Culex* (*Culex*) *pipiens* L. 1758 and *Culex* (*Culex*) *torrentium* Martini, 1925, respectively, and 241 larvae (18.7 %) as *C. hortensis*. The proportion of Anopheles Claviger Complex larvae was 0.5 %.

Associated faunal taxa in the rock pools were Cladocera, *Asellus aquaticus*, *Velia* sp., *Hydrometra gracilis*, *Laccobius striatulus* (Dytiscidae), Dryopidae, Chironomidae (e.g. *Tanypus* sp.), Tipulidae, Trichoptera, Ephemeridae, Plecoptera and *Bombina variegata*.

Three plant species were found: *Hygroamblystegium fluviatile*, *Brachythecium rivulare* and filamentous algae (*Spirogyra* sp.).

### Abiotic parameters of the breeding sites

The rock pool breeding sites were characterised by an alkaline pH, low conductivity and low values of hardness. The values for ammonium, nitrite, nitrate and phosphate were either below the limits of detection or very low (Table 1).

**Table 1:** Abiotic parameters of the rock pools in the Murg and the floodplains of the river Rhine

Sampling site	Murg (rock pools)	Rhine floodplains
pH	8.35	7.02
Conductivity [μS]	30	690
Total hardness [dH°]	2.4	19.7
Carbonate hardness [dH°]	2.5	11.6
Ammonium [mg/l]	<0.2	<1
Nitrite [mg/l]	0	25
Nitrate [mg/l]	0	<10
Phosphate [mg/l]	0	<0.5

### Diagnostic morphological characters of *An. petragnani* and *An. claviger* s.s. larvae

**Fig. 2:**
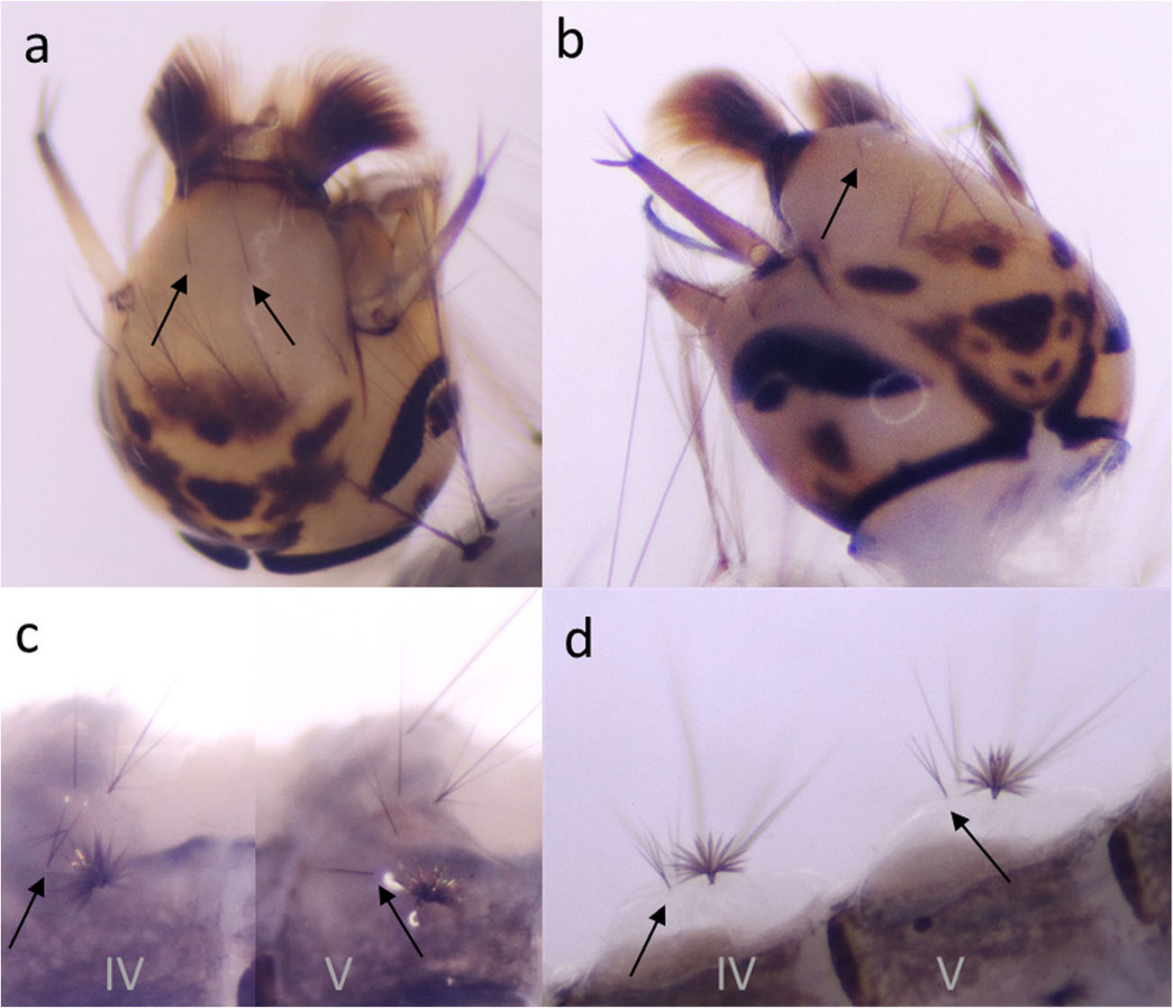
Diagnostic setae of *An. claviger* s.l. larvae. **a** Postclypeal setae of *An. petragnani*. **b** Postclypeal setae of *An. claviger* s.s. **c** Antepalmate setae of abdominal segments IV and V of *An. petragnani*. **d** Antepalmate setae of abdominal segments IV and V of *An. claviger* s.s.

The number of branches of the postclypeal setae (4-C) of the larvae of *An. petragnani* ranged from one to two, but was usually one. When two branches were counted, the second was much smaller than the main one. The number of antepalmate seta branches (2VI and 2V) ranged from two to three, but in general was two (Table 2, Fig. 2).

**Table 2:** Number of branches of postclypeal and antepalmate setae for the three populations of *An. claviger* s.l

Postclypeal setae (4-C) no. of branches	1	2	3	4	5	6
*An. claviger* s.s. (R)	4	13	20	2		
*An. claviger* s.s. (M)		1	3			
*An. petragnani* (M)	6	2				
Antepalmate setae (2-IV + 2-V) no. of branches	1	2	3	4	5	6
*An. claviger* s.s. (R)			21	36	22	1
*An. claviger* s.s. (M)			1	2	3	2
*An. petragnani* (M)		13	2			

Provided are the numbers of counted setae with the corresponding numbers of branches per seta. *An. claviger* s.s. Neuried: 20 individuals; *An. claviger* s.s. Murg: 2 individuals; *An. petragnani* Murg: 4 individuals. Setae not counted were absent (*R* Rhine, *M* Murg)

In *An. claviger* s.s., the number of branches in the postclypeal setae was usually two to three, but ranged from one to four. The number of antepalmate seta branches ranged from three to six, but three to five were usually counted.

### Determination of *An. claviger* s.l. by PCR-based diagnostics

**Fig. 3:**
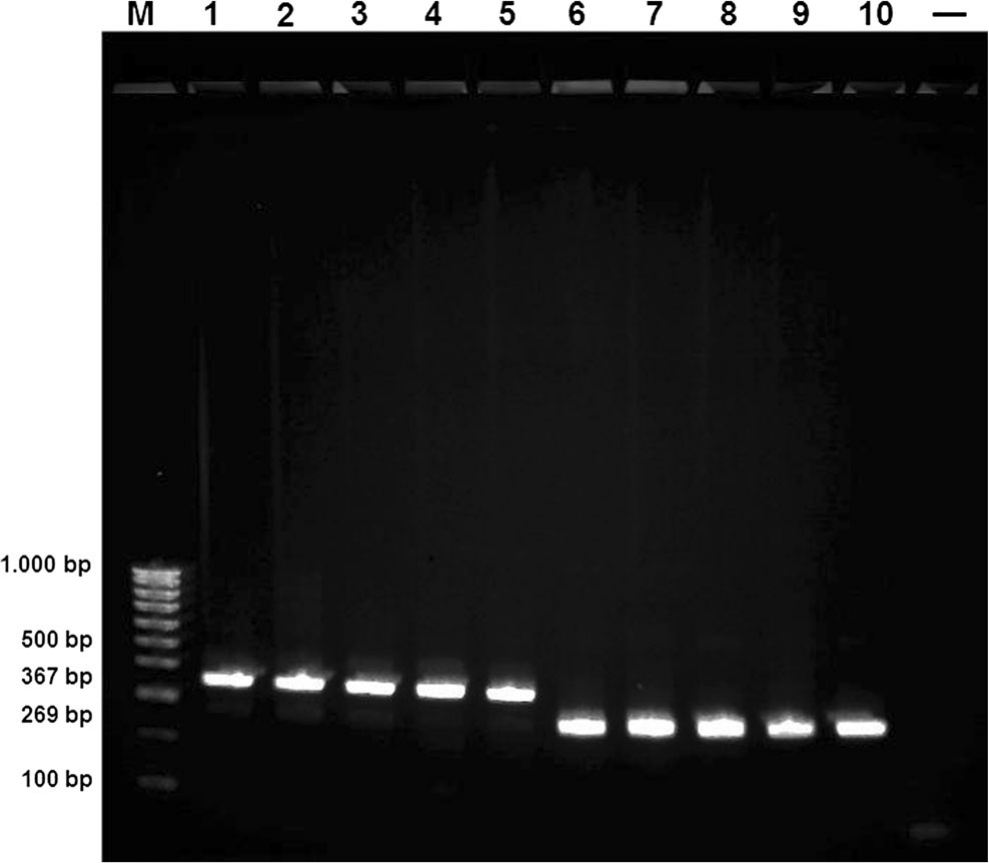
Species diagnostic internal transcribed spacer 2 (ITS2) fragments from all *An. petragnani* (367 bp) and some *An. claviger* s.s. (269 bp) individuals found during this study (*M*, Quantitas DNA Marker 100 bp–1 kb, Biozym; *lanes 1–5*, *An. petragnani*; *lanes 6–10*, *An. claviger* s.s.; − negative control)

Individuals belonging to the Anopheles Claviger Complex (altogether 22 samples) were further investigated using a species-specific PCR (Kampen et al. 2003; Sternberg 2004) to verify the morphological classification. Five samples showed positive signals for *An. petragnani*, the remaining 17 for *An. claviger* s.s. Figure 3 illustrates the species-specific PCR fragments after agarose gel electrophoresis of 10 samples, of which five were *An. petragnani* (from the Black Forest, 367 bp) and five *An. claviger* s.s. (269 bp, samples 6 and 7 originating from the Black Forest, samples 8–10 from the Rhine floodplain).

The sequencing of all amplificates confirmed the results of both the morphological classification as well as the PCR by blasting the obtained sequences in NCBI and comparing them to the already inscribed sequences of *An. petragnani* (accession number GenBank AY129233.1) and *An. claviger* s.s. (accession number GenBank AY129232.1).

**Fig. 4:**
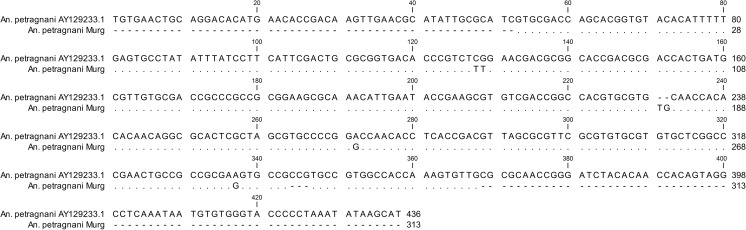
Alignment of the *An. petragnani* consensus sequence obtained during this study with the *An. petragnani* consensus sequence published under AY129233.1 in GenBank

The alignment of all the sequences is shown in Fig. 4. The Murg *An. petragnani* ITS2 sequence revealed three SNPs and two indels compared to the consensus sequence from the Mediterranean samples published in GenBank.

## Discussion

This is the first time that *An. petragnani* has been recorded north of the Alps. So far, this species was only found in the western Mediterranean area in Italy, including Sardinia (Coluzzi et al. 1965; Marchi and Munstermann 1987; Romi et al. 1997), France including Corsica (Coluzzi et al. 1965; Schaffner 1998), Spain (Encinas Grandes 1982; Eritja et al. 1998), Portugal (Pires et al. 1982; Ramos et al. 1977; Ribeiro et al. 1988; Ribeiro et al. 1996) and North Africa (Coluzzi et al. 1965; Senevet and Andarelli 1955). Coluzzi (1962) found *An. petragnani* to be a more thermophilic species than *An. claviger* s.s. Therefore, it is surprising to find *An. petragnani* outside of its previously known range in the Mediterranean climate. Based on published distribution data, it appears to be an isolated population in the mountains of the Black Forest. Despite intense research, it could not yet be found in the neighbouring Vosges and most parts of France, except for the known southern regions (Kampen et al. 2003; Schaffner et al. 2003).

In contrast to *An. petragnani*, *An. claviger* s.s. is distributed all over Europe, extending east towards Afghanistan and south into North Africa (Ramsdale and Snow 2000). *An. claviger* s.s. is more widely distributed in northern Europe and exploits cold water issuing from springs, wells and underground cisterns in Mediterranean regions (Coluzzi 1962).

For differentiating the sibling species, we used the chaetotaxy described by Coluzzi (1960) as well as a species-specific PCR-based diagnostic assay (Kampen et al. 2003; Sternberg 2004). The combination of morphological and molecular-based determination techniques allowed us to distinguish reliably between the two species and enabled us to review the variability and slight overlap in the number of diagnostic setae of larval stages. Reliable species discrimination is essential for the analysis of the bionomics of *An. claviger* s.s. and *An. petragnani*, such as preferred breeding sites, specific biting behaviour and vector competence, particularly for malaria parasites. According to the literature, it seems that *An. claviger* s.s. has a much broader ecological range than *An. petragnani*. So far, the latter was found in freshwater rock pools, ditches, drainage canals, edges of rivers and streams (Marchi and Munstermann 1987), but also in artificial breeding sites like wells and cisterns (Romi et al. 1997).

Exotic species, such as *Ae. j. japonicus* or *Aedes* (*Stegomyia*) *albopictus* (Skuse, 1895), are usually introduced by the transportation of desiccation-resistant eggs (Becker et al. 2011; Pluskota et al. 2008). This is unlikely for anopheline species, which usually lay their eggs directly on the water surface. How the breeding sites in the Black Forest were colonised by *An. petragnani* so far away from its known distribution area is of particular zoogeographic interest. A recent introduction of *An. petragnani* to the Murg valley seems unlikely, as the ITS2 sequence differentiation between the Mediterranean *An. petragnani* and the Murg population is considerable, consisting of three SNPs and two indels. This differentiation indicates a discontinuous geographical distribution without gene flow due to isolation by distance. Based on this assumption, the Murg population might be the relict of a warmer postglacial period in the early Holocene, when the distribution of *An. petragnani* probably extended further north and maybe was not fragmented.

Also minor differences in the number of branches in the antepalmate setae of the Murg and Mediterranean *An. petragnani* populations could be found. However, this observation is based only on a small sample size. In the four larvae examined in this study, two branches were usually counted, whereas Coluzzi reported three branches to be common (Coluzzi 1960).

**Fig. 5:**
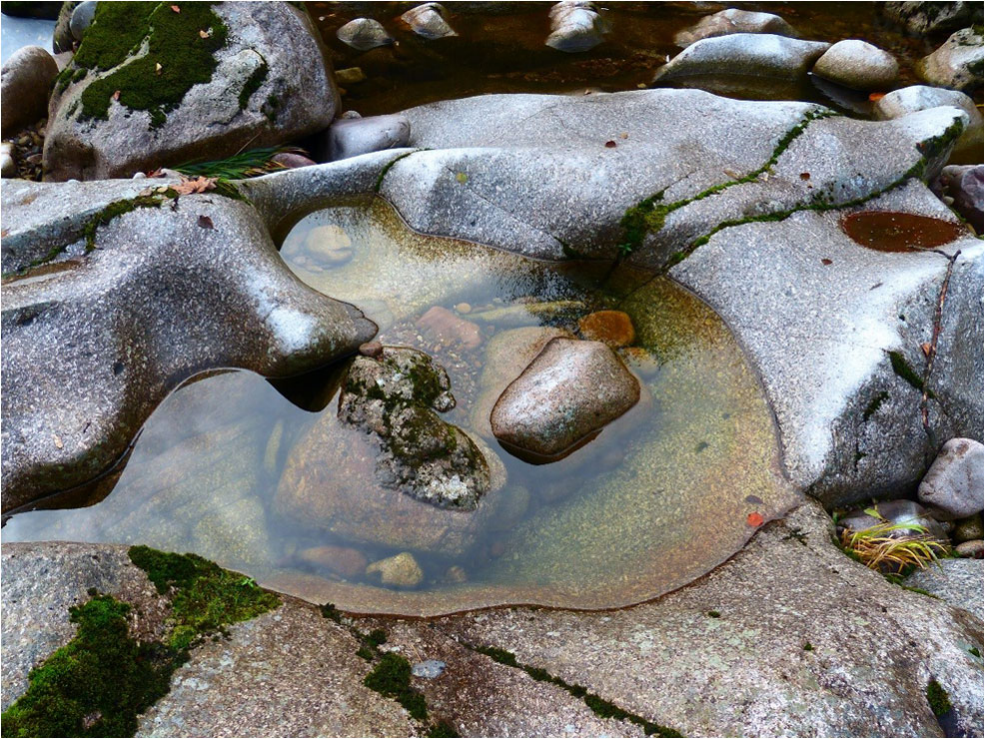
Rock pool in the granite river bed of the Murg

In the Murg rock pools, little vegetation was present, and only the water mosses *H. fluviatile* and *B. rivulare* as well as filamentous algae were present. It remains unclear whether the occurrence of water mosses is important for the breeding site choice and egg deposition of the anopheline females (Pires et al. 1982). Anopheline larvae could be found in breeding sites where the water moss lined the edges of the water bodies and never in potential breeding sites consisting of pure granite (Fig. 5).

This finding of *An. petragnani* is the seventh record of a so far unknown or newly introduced mosquito species in Germany since the middle of the 1990s, alongside *Uranotaenia* (*Pseudoficalbia*) *unguiculata* Edwards, 1913 (Becker and Kaiser 1995), *Ae. albopictus* (Becker et al. 2013; Pluskota et al. 2008), *Ae. j. japonicus* (Becker et al. 2011; Schaffner et al. 2009), *Culiseta* (*Allotheobaldia*) *longiareolata* (Macquart, 1838) (Becker and Hoffmann 2011), *Anopheles* (*Anopheles*) *daciae* Linton, Nicolescu & Harbach 2004 (Weitzel et al. 2012) and *Aedes* (*Finlaya*) *koreicus* (Edwards, 1917) (Werner et al. 2015).

Further research is required to define the occurrence of both sibling species of the Anopheles Claviger Complex, with a focus on niche breeding sites in temperate regions, like rock pools, that could be favourable for *An. petragnani*. This would also answer the question whether *An. petragnani* in the Black Forest is an isolated population. Sequencing of the complete ITS2 region and analysis of potential geographic variation in nucleotide sequences could provide further information on the origin of the German *An. petragnani* population and the dispersal of both *An. petragnani* and *An. claviger* s.s. as a result of climate variation and space-time processes.
